# Seismic performance of the replaceable steel links with different short length ratios

**DOI:** 10.1038/s41598-024-81336-8

**Published:** 2024-12-02

**Authors:** Liquan Xiong, Zhengchao Guo, Jun Cai, Kaiyu Jiang, Linyan Li

**Affiliations:** 1 Department of Civil Engineering, Chongqing Three Gorges University, Wanzhou, Chongqing, 404100 China; 2 Chongqing Engineering Research Center of Disaster Prevention & Control for Banks and Structures in Three Gorges Reservoir Area, Wanzhou, Chongqing, 404100 China

**Keywords:** Replaceable steel links, Quasi-static test, Seismic behavior, Shear strength, Finite element analysis, Mechanical engineering, Civil engineering

## Abstract

Compared with the traditional frame structures, the novel composite frame with replaceable steel links can realize the rapid recovery of building occupancy after seismic actions to provide the capacity of reducing structural damage and ensure the reuse of a structure. In this system, plastic deformation and damage mainly concentrated in the steel links serving as the structural fuses and other structural members still remained elastic or minor plastic, and then the damaged steel links can be easy for replace. In order to study the seismic performance of the replaceable steel links, a total of four test specimens with different short length ratios were designed. A low-cycle reversed loading test was carried out in terms of failure characteristic, hysteretic response and shear strength. The results indicated that all tested specimens presented a stable and full hysteretic response while the steel links especially exhibited a large inelastic deformation capacity and fully developed shear strength as the structural fuses. Besides, failure modes of steel links included two types of shear failure and bending-shear failure, followed by two types of damage features, including web-to-stiffener weld tear, web buckling, flange-to-end plate weld tear and flange buckling, respectively. Finally, nonlinear finite element models (FEMs) of test specimens were implemented by the Abaqus software. The numerical results were in good agreement with the experimental results in terms of load-deformation curves, initial stiffness, the development of Von Mises stress and cumulative plastic equivalent strain (PEEQ) indexes to facilitate design practice.

## Introduction

According to the current earthquake resistance design code for the buildings, the traditional structural systems can achieve the predetermined levels of no damage under small earthquake, repairable under moderate earthquake and no collapse under major earthquake. However, the concentrated damage elements consisted of beams, columns, beam-column joints and walls to undergo inelastic deformation and dissipate seismic energy during the earthquake might cause the nonductile failure and their post-damage repair that is expensive and time-consuming abundantly, and even to demolish and reconstruct. The loss of occupancy and difficulties of repairing the damaged system economically burden the owners and occupants in the society. Correspondingly, earthquake resilient structure systems with the capacity of rapidly returning to occupancy following an earthquake have attracted increasing interest as one of the frontiers in the earthquake engineering to solve these of problems in practice^1–3^.

Therefore, seismic design philosophy of the structure systems has generally shifted from preventing structural collapse to restore the structure function^4,5^. Earthquake resilient structure systems have been arising as a cost-effective alternative to the traditional steel or reinforced concrete frame structures^6,7^because of the superior system characteristics of limited structural damage and residual displacement, and rapid return to occupancy quickly after the earthquake. According to this philosophy, damage of earthquake resilient structures focused on the replaceable members or other concentrated structural components by these mechanisms of replacing, self-centering, rocking and energy-dissipating device^8^. Extensive studies^6–11^ have indicated that earthquake resilient structures generally consisted of frame systems, eccentrically braced frames, RC coupled wall systems, buckling-restrained braced frames and linked column frames. Among of these structures, the systems with replaceable members have been popular accepted recently. The main reason is that the plastic deformation and damage mainly focused on the replaceable links serving as the structural fuses and other structural members still remained elastic or minor plastic, and then the damaged links can be easy for replace with new links to quickly achieve a seismic rehabilitation after a major earthquake.

In order to evaluate the seismic behavior and replacement capacity of the replaceable shear links after the earthquake, various types of the replaceable links and linked connections in different systems have proposed by the engineers and scholars^7–15^, such as end plate bolted connection, web bolted connection, T-shaped bolted connection and so on. Extensive studies have indicated that the factors consisting of length ratio, section form, shear span-to-depth ratio, axial force, stiffener layout and beam-to-link strength ratio have a significant effect on the seismic performance of the replaceable links^14–21^. Among these parameters, the length ratio played a major role in the failure feature and energy dissipation capacity of the replaceable links. Note that the length ratio *ρ* of the replaceable links is *e*/(*M*_*p*_/*V*_*p*_), where *e* denotes the linked steel length, *M*_*p*_ and *V*_*p*_denote the plastic flexural strength and shear strength according to the actual measured yield strength of the actual measured dimensions of steel links, respectively. From the previous findings of Popov and and his colleagues^22^, the replaceable links with the length ratios *ρ* < 1.6 presented the shear dominated behavior. In addition, the replaceable links with length ratios of 1.6 < *ρ* < 2.6 classified as intermediate replaceable links displayed the shear-flexure dominated behavior, while the replaceable links with *ρ*> 2.6 generally referred to as long links showed the flexure dominated behavior. This proposed recommendation has been adopted by many design specifications such as AISC 341–16^23^and CMC GB 50,011–2017^24^.

Compared with the traditional RC coupled wall systems with replaceable link beams and eccentrically braced frames with replaceable link beams, the proposed linked column frames with replaceable steel links consisting of traditional frames, two steel columns and steel shear links presented the differences of structural feature and axial constraint of link ends. Additionally, because of achieving rapid return to occupancy under predetermined levels of lateral load, the linked column frame with closely spaced bay was generally required to increase the lateral stiffness and attract maximum seismic force under major seismic loading. In this context, there is a clear need to estimate the behavior and replaceability of steel links that could facilitate the disassembly and replacement under different stages. Characteristics of linked column frames with replaceable steel links were first introduced. The next section of this paper describes an experimental program where four steel links with different short length ratios were subjected to the cyclic loading. The experimental results were then detailed in terms of failure mode, hysteretic response and shear strength. Finally, nonlinear 3D finite element models using ABAQUS 6.14^25^ were developed and validated to capture the observed experimental responses.

## Characteristics of linked column frames with replaceable steel links

**Fig. 1:**
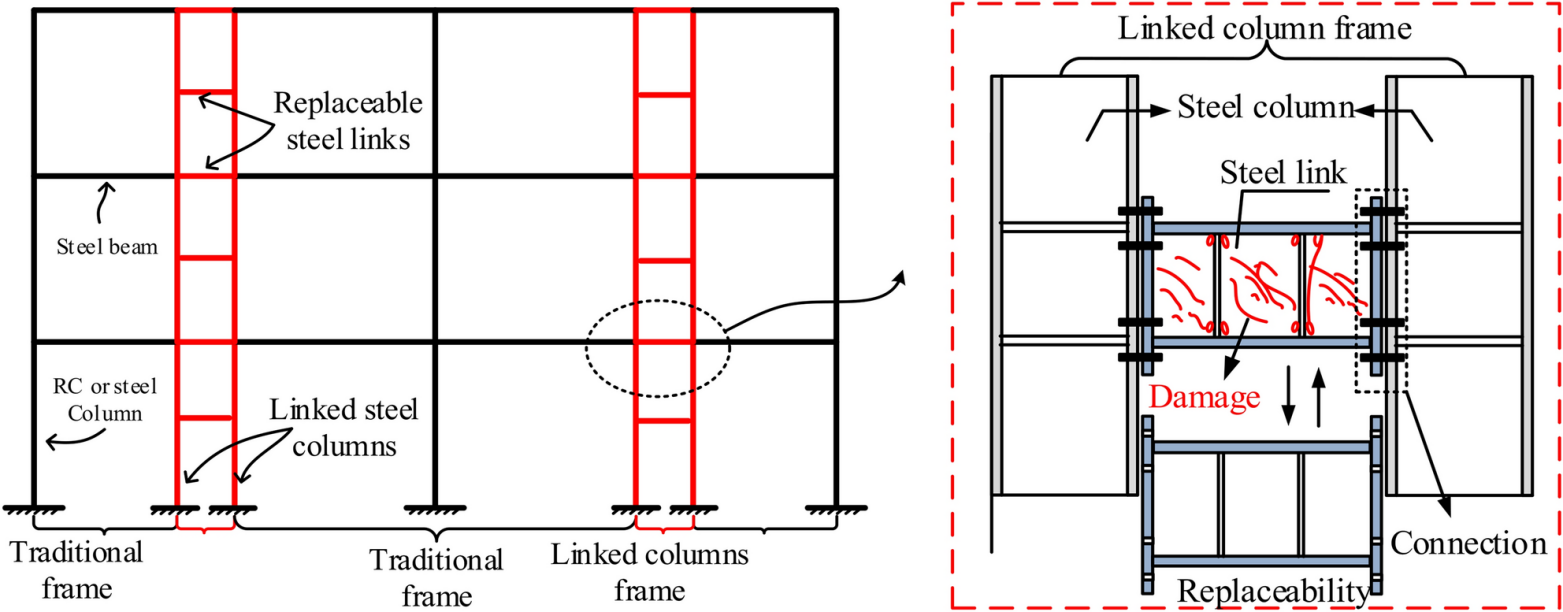
Schematic diagram of linked column frames with replaceable steel links.

Figure 1 depicts characteristics of linked column frames with replaceable steel links to improve the seismic performance compared to these of the traditional steel or steel–concrete composite frame structures. As shown in Fig. 1, this proposed structural system consisted of traditional steel or steel–concrete composite frames and linked column frames. The steel links specialized by those of dismountable connections^8^, such as extended end plate, slotted bolted end plate and bolted web, was generally positioned at the floor or floor and inter-storey level, depending on the desired degree of coupling for the composite frame structure. The target of dual steel columns interconnected with replaceable steel links mainly provides lateral stiffness and inelastic behavior to the system by having the links yield primarily in shear under predetermined levels of lateral load, while the traditional frame provides the primarily gravity load-carrying. This composite frame structure with replaceable steel links can realize the rapid recovery of building occupancy after seismic actions such that this system possesses the capable of reducing structural damage and ensuring continued use of the structure. Under the predetermined earthquake, the plastic deformation and damage in this systems mainly concentrated on the steel links serving as the structural fuses and other structural members still remain intact or minor damage, and the damaged steel links can be easy for replace to achieve the recovery of functionality.

For this system, two key parameters of the elastic stiffness ratio *K*_*LF*_/*K*_*MF*_ and yield displacement ratio* Δ*_*MF*_/*Δ*_*LF*_are used as two control parameters for the elastic design to assist with self-centering and replacement operations according to the previous findings of Men et al. (2020, 2021)^26,27^and Malakoutian (2012)^28^. Where *K*_*LF*_ and *Δ*_*LF*_ donate the elastic stiffness and yield displacement of the linked columns frame, respectively; *K*_*MF*_ and *Δ*_*MF*_ are the elastic stiffness and yield displacement of the traditional composite frame or steel frame, respectively. The FEM results showed that the composite structure can achieve the predetermined levels with good replaceability and the dual-parameter seismic design method should be effective when the factor *K*_*LF*_/*K*_*MF*_ is in the range of 2.8 to 4.3 and the factor *Δ*_*MF*_/*Δ*_*LF*_ is greater than 1.8.

**Fig. 2:**
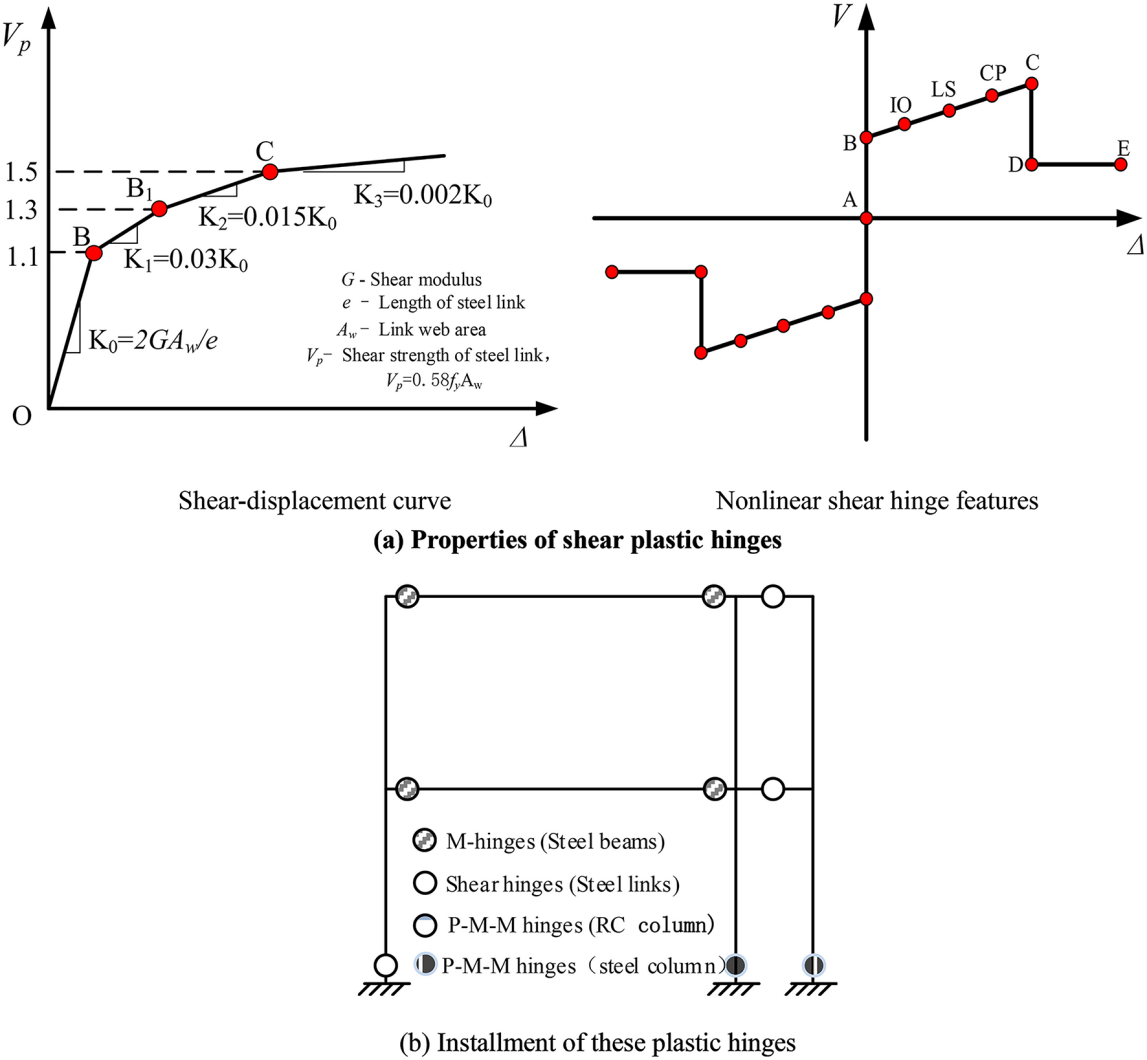
Properties of these shear and moment plastic hinges in this model

In light of the above discussions, nonlinear analysis was conducted using SAP2000 software under the pushover analysis. An 3-story, 10.8-m tall building with the plan dimension of 30.0 × 16.5 m was modeled. The span of RC column is 9.0 m and that of linked columns is 1.0 m, respectively. And the steel links were only installed at floor. Vertical schematic of the linked column frame is similar to that of composite frame with replaceable steel links, as shown in Fig. 1. The sections properties of RC column, steel beam, linked column and linked beams are 600 × 600 mm, H350 × 180 × 8 × 12 mm, H400 × 400 × 16 × 25 mm and H400 × 200 × 10 × 18 mm, respectively. And material properties of these corresponding structural members included concrete grade of C40, steel grade of Q235, steel grade of Q355 and Q235 for steel web and Q355 for steel flanges were adopted, respectively. Additionally, Fig. 2 (a) shows the strength degradation of shear-rotation behavior of plastic hinges was modeled among the steel shear link ends. As shown in Fig. 2 (b), plastic of these moment hinges was installed at the ends of traditional steel beams and these of P-M-M hinges were adapted to the end of the RC columns and linked steel columns, respectively. P-Delta effects and the gravity loads during the pushover assessment should be considered in the modeling. Note that fatigue strength of steel material and deconstruc- tability of steel links were not investigated in this study.

**Fig. 3:**
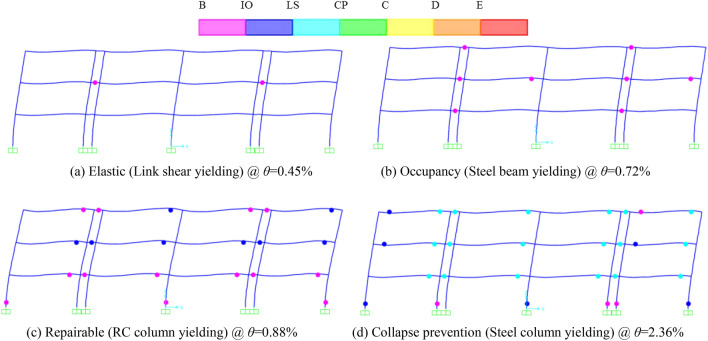
Yield stages of structure members of the 3-story frame

Figure 3 shows these plots of deformed shape and plastic hinge formation with the maximum story drift in the linked column frames with replaceable steel composite frame. Where B denotes the yield of hinge for these structural members, IO denotes the Immediate Occupancy, LS denotes the Life Safety, CP denotes the Collapse Prevention, C denotes the Collapse stage, *θ* denotes the maximum top story drift of the whole composite system, respectively. The yield process of members in this structure displays the yield mechanism of the structure as well. As observed in these graphs, first all steel links yield, and then the flexural steel beams enter the inelastic phase and undergo the permanent deformations, and the columns yield finally. Particularly, the structural members remain elastic before the yield of steel links (Fig. 3(a)). As illustrated in Fig. 3(b), the plastic deformations mainly concentrate in the replaceable steel links to protect other structural components, and then the damaged links can be easy replaced with the new links to rapid return to occupancy under predetermined levels of lateral load. Moreover, the steel links continue to dissipate energy and these steel beams form the moment plastic hinges to be repaired (Fig. 3(c)). Finally, the whole system presents the anti-collapse performance level (Fig. 3(d)). Therefore, this proposed system with replaceable steel links can provide for four performance levels: elastic, rapid return to occupancy, repairable and collapse prevention under earthquake. In addition, the structures can improve the seismic performance of the traditional system and realize the seismic function of the post-earthquake resilience. Note that the roof drift ratio differences between the yield points of steel links and traditional steel beams represents the rehabilitation capacity of this composite system. Besides, the process of damage features performed that the performance of this composite system is agreed with these of the previous findings of Men et al. (2020)^27^and Shoeibi et al. (2018)^29^. One of the key factor in this system achieving the desirable seismic performance is whether the replaceable steel links acts as a “fuse” member or not to design for the lateral seismic demands. Therefore, there is a clear need to investigate the adequacy of the replaceable steel links by increasing their stiffness, load carrying capacity, fatigue strength and deconstructability.

## Experimental program

### Test specimens

As discussed before, a 3-story composite frame structure with replaceable steel links was designed as the prototype structure referring to Chinese Standard GB 50,017–2017^24^and ANSI/AISC 341–16^23^. The second story of the prototype structure was selected as the representative story for quasi-static cyclic tests based on the laboratory conditions. Additionally, simple hinge supports were defined for the top and bottom of the half steel columns to simulate the inflection points under lateral loading.

**Fig. 4:**
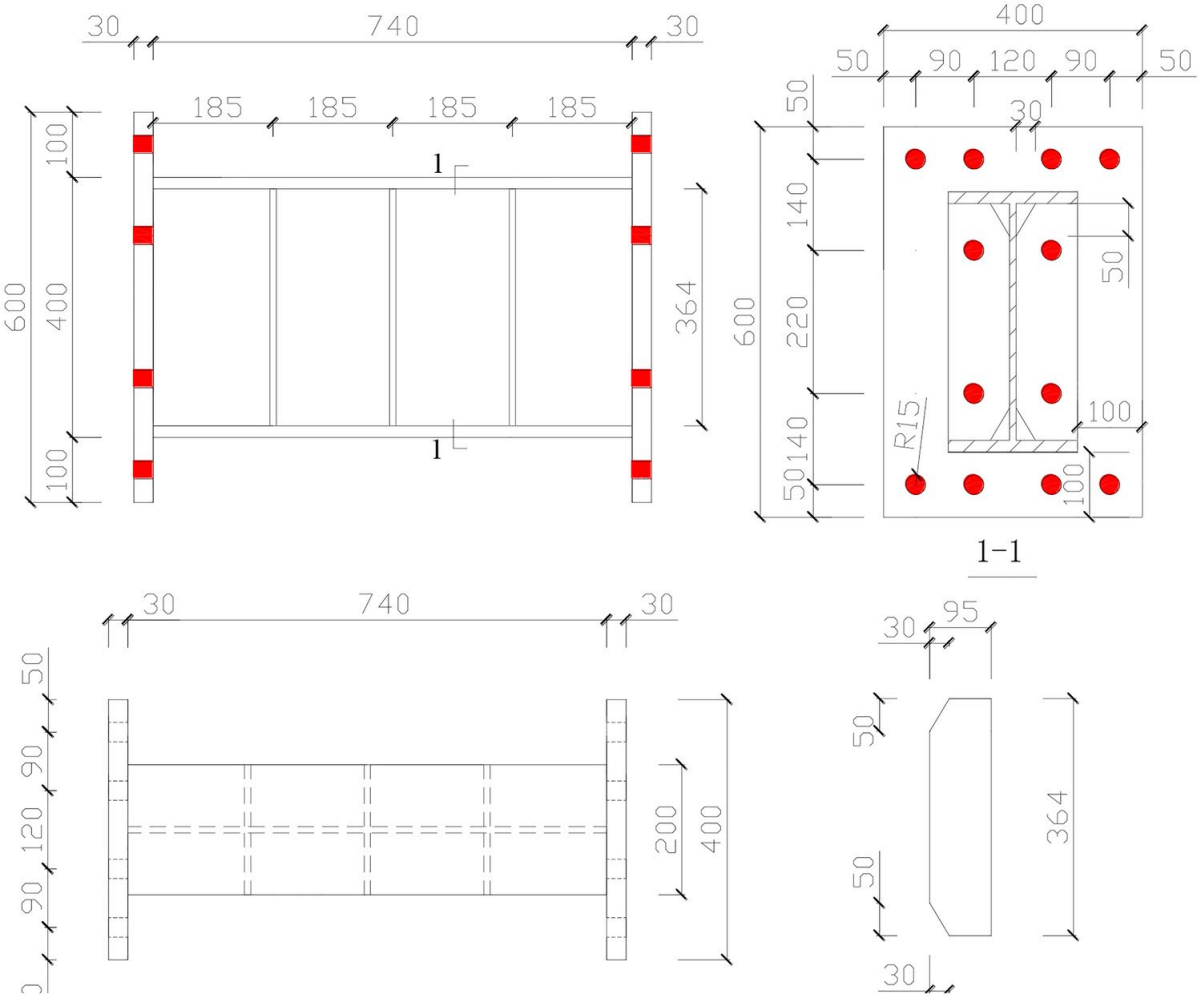
Dimension of specimen RL1 ( unit: mm).

To evaluate the seismic behavior of the replaceable steel links, a total of four steel links were considered in this test. Figure 4shows the geometric configurations and dimensions of specimen RL1. Other three specimens are similar with that of specimen RL1 except for the link length. All replaceable steel links adopted the hybrid sections. The cross sections of all specimens were built-up H-shapes with H400 × 200 × 10 × 18 mm and the width-to-thickness ratio of the flange and the web were 5.2 and 36.4, respectively. Those of which were satisfied the recommendations for the BEFs links by the AISC 341–16 (AISC 2016)^23^and CMC GB 50,011–2017^24^. The link specimens with short length ratio had a length ratio smaller than 1.6, and therefore were expected to yield primarily in shear per the AISC 341–16 (AISC 2016)^23^provisions. Emphatically, extensive studies^6–8,30–32^have indicated that the steel links with short length ratios can provide a better strength, inelastic deformation and excellent energy dissipation capacity under cyclic loading. Combined with seismic design philosophy of this composite frame structure with replaceable steel links, the steel links serving as the structural fuses mainly provide inelastic behavior to the system by having the links yield primarily in shear under predetermined levels of lateral load. The lengths of all links were 740, 940, 1140 and 1740 mm and those length ratios of specimens were 0.68 and 0.86, 1.05 and 1.60 to realize the shear dominated behavior, respectively. To dismount or reinstall the links after damage, referring to the design philosophy of the connection by Sumner (2003)^33^, the end-plate connections of replaceable steel links between the link and column were designed. For the replaceable steel links, the full-depth stiffeners of web utilized on both sides and the average distance satisfied the limit value (30*t*_*w*_ – *h*/5) to prevent premature local web buckling, where *t*_*w*_ denotes the steel web thickness and *h* denotes the steel web depth. In addition, the intermediate web stiffeners from the toe of web-to-flange weld were partly cut off to alleviate the stress concentration on the fillet welds affected zone at the junction of the flanges and web. The characteristics of each steel links are depicted in Table 1.

**Table 1:** Features of RL specimens.

Specimen number	Length *e* (mm)	Section of specimen		Length ratio *e*/( *M*_*p*_/*V*_*p*_)	Stiffener spacing (mm)
		Yield bend *M*_*p*_ (kN.m)	Yield shear *V*_*p*_ (kN)		
RL1	740	538.5	496.1	0.68	4@185
RL2	940			0.86	5@188
RL3	1140			1.05	6@190
RL4	1740			1.60	8@218

The two steel linked columns with the section of H600 × 400 × 18 × 25 mm were reused and the material is Q345 grade steel. The flange of the replaceable links was Q345 grade steel, and the web was Q235 grade steel. Table 2 shows the mechanical properties of the steel by tensile coupon tests.

**Table 2:** Mechanical properties of steel.

Type of steel	Plate	Thickness t / mm	Yield strength *f*_*y*_ (MPa)	Tensile strength *f*_*u*_ (MPa)	Yield strength ratio *f*_*u*_ / *f*_y_	Yield strain *ε*_*y*_ (*με*)	Elastic modulus *E*_s_ (× 10^5^ MPa)	Elongation *δ/%*
Q235	Web	10	291.7	441.7	1.51	1383	2.11	41.5
Q345	Flange	18	391.7	538.3	1.37	1967	1.99	42.5

### Test setup and loading protocol

**Fig. 5:**
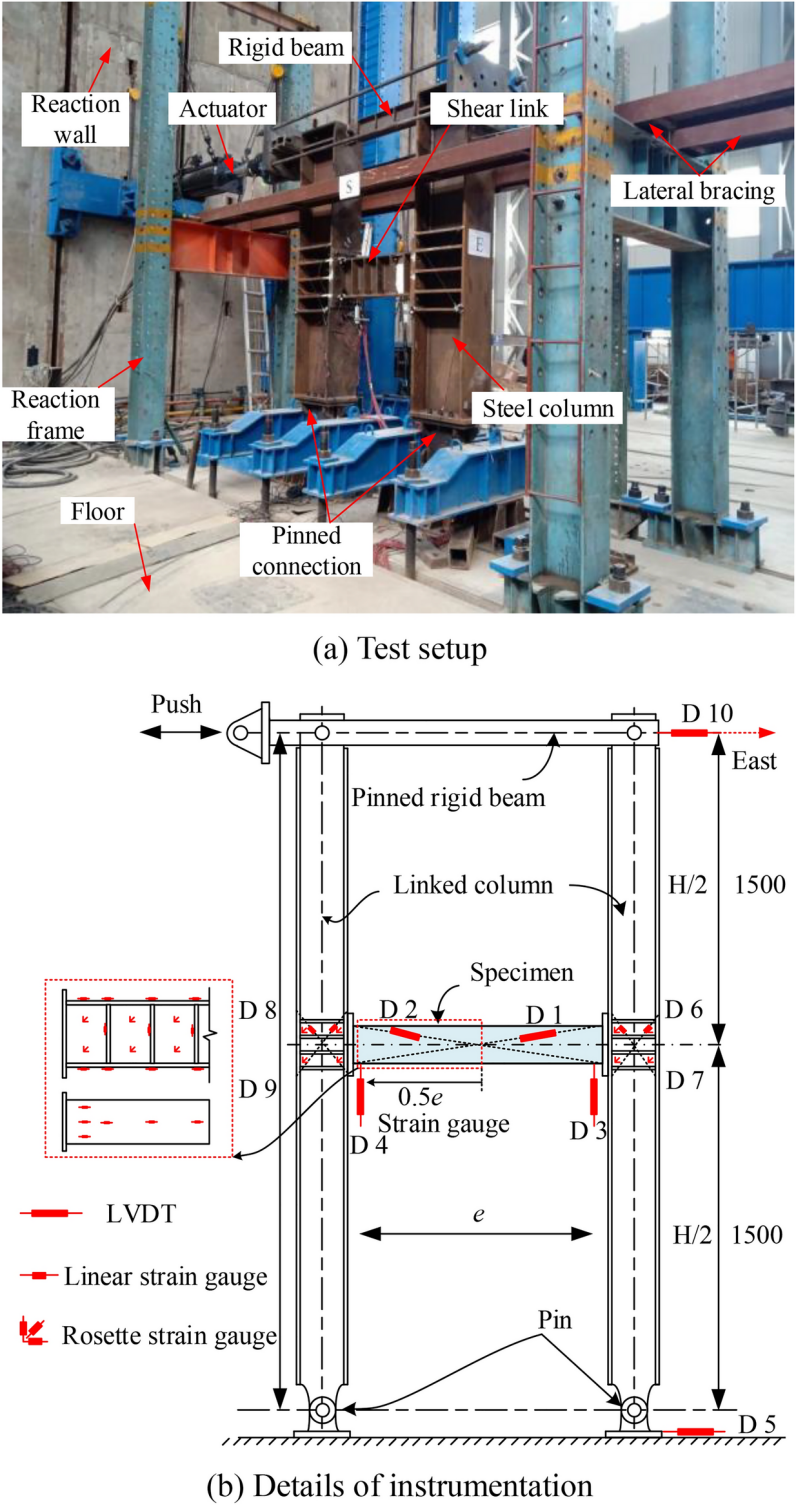
Test setup and and instrumentation (unit: mm).

Figure 5 illustrates the test setup and details of instrumentation. A 1000 kN hydraulic actuator attached to the steel column ends was applied the reversed lateral displacement, as shown in Fig. 5(a). The linked steel column was pinned to the ground. The two tops of the steel column were connected by a rigid beam and the end of the replaceable steel links was also connected to the steel column by the end plate bolted connections. In order to ensure the lateral stability of the tested specimen during the loading, lateral bracing was used along the column flanges. Displacement transducers and strain gauges were installed to measure the displacement and strain for RL specimens and columns. And some diagonal displacement transducers also installed to estimate the rotation deformations of the shear link and the link-to-column connections. To measure the strains in the link web and flanges and the steel column, linear and rosette strain gauges were used in the test, as displayed in Fig. 5(b). According to the previous findings of Men et al.^8,13^and Ji et al^8,18^., the shear force and rotation deformations of the replaceable steel links in this test can be measured.

**Fig. 6:**
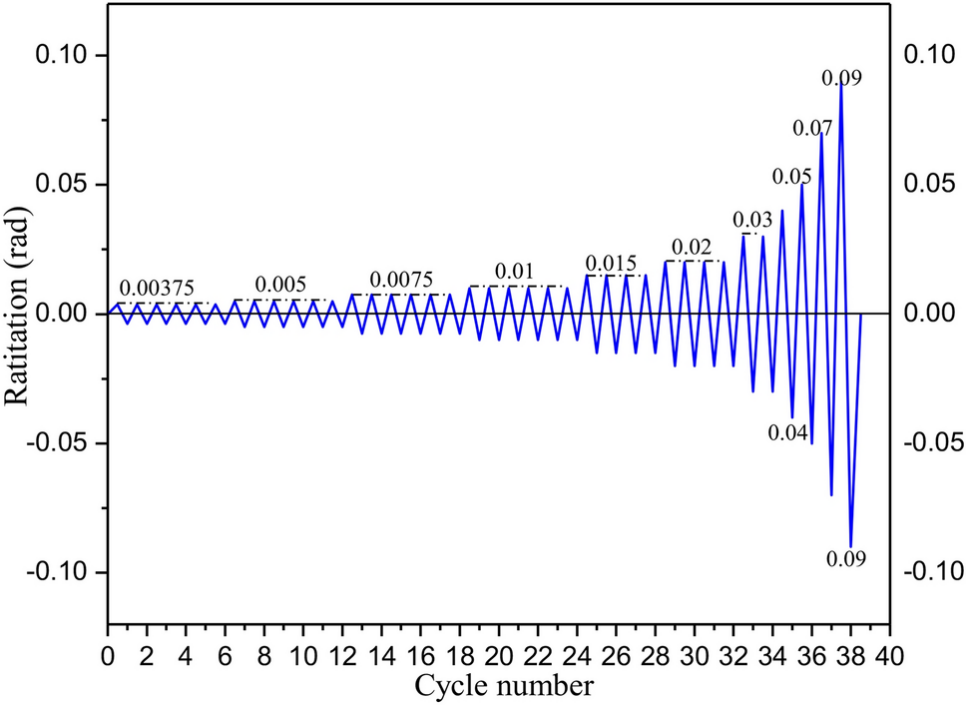
Loading protocol.

Figure 6shows the displacement-controlled loading protocol for the test, referring to the American guidelines of AISC 341–16 (AISC 2016)^23^. The initial loading displacement amplitude was 0.00375 rad, 0.005 rad, 0.0075 rad and 0.01 rad for six cycles at each amplitude; and then the loading displacement amplitude was 0.015 rad and 0.02 rad for four cycles. Subsequently, two cycles at 0.03 rad and one cycle at 0.04 rad and 0.05 rad were reached, respectively. Finally, increased in increments of 0.02 rad for one cycle until the specimens reached failure was applied.

## Experimental phenomena and failure modes

### Failure characteristics of the specimens

**Fig. 7:**
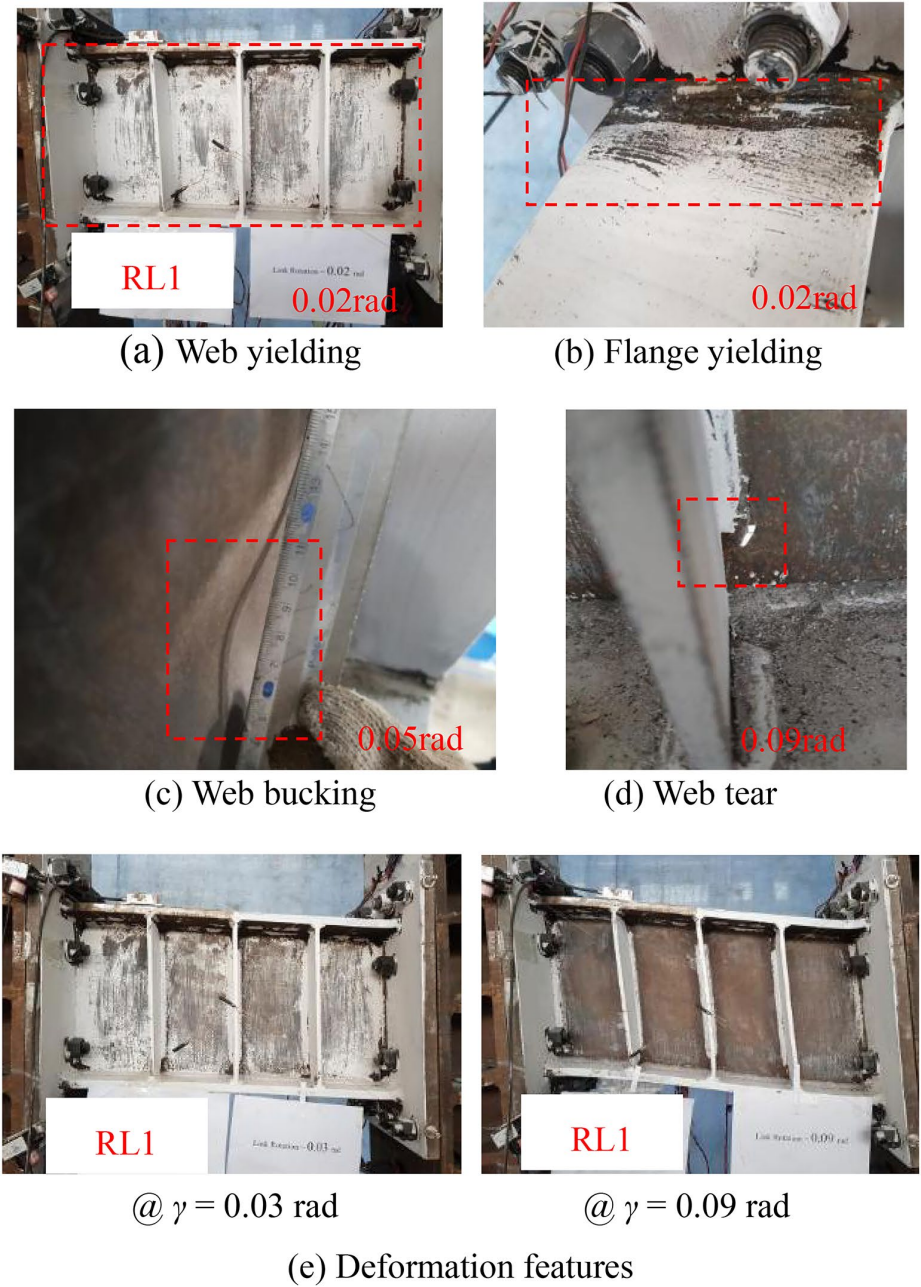
Test phenomena of specimen RL1.

**Fig. 8:**
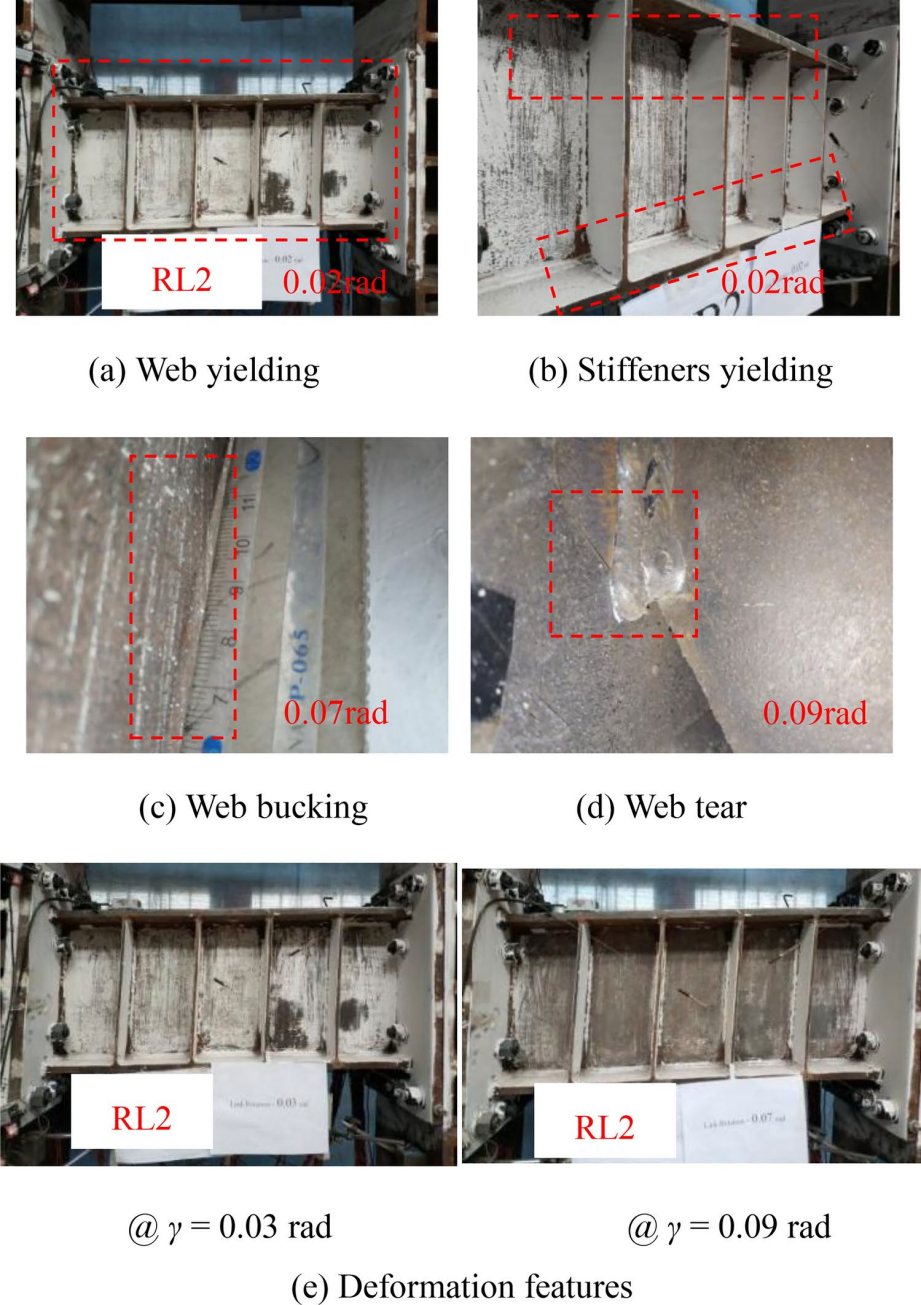
Test phenomena of specimen RL2.

**Fig. 9:**
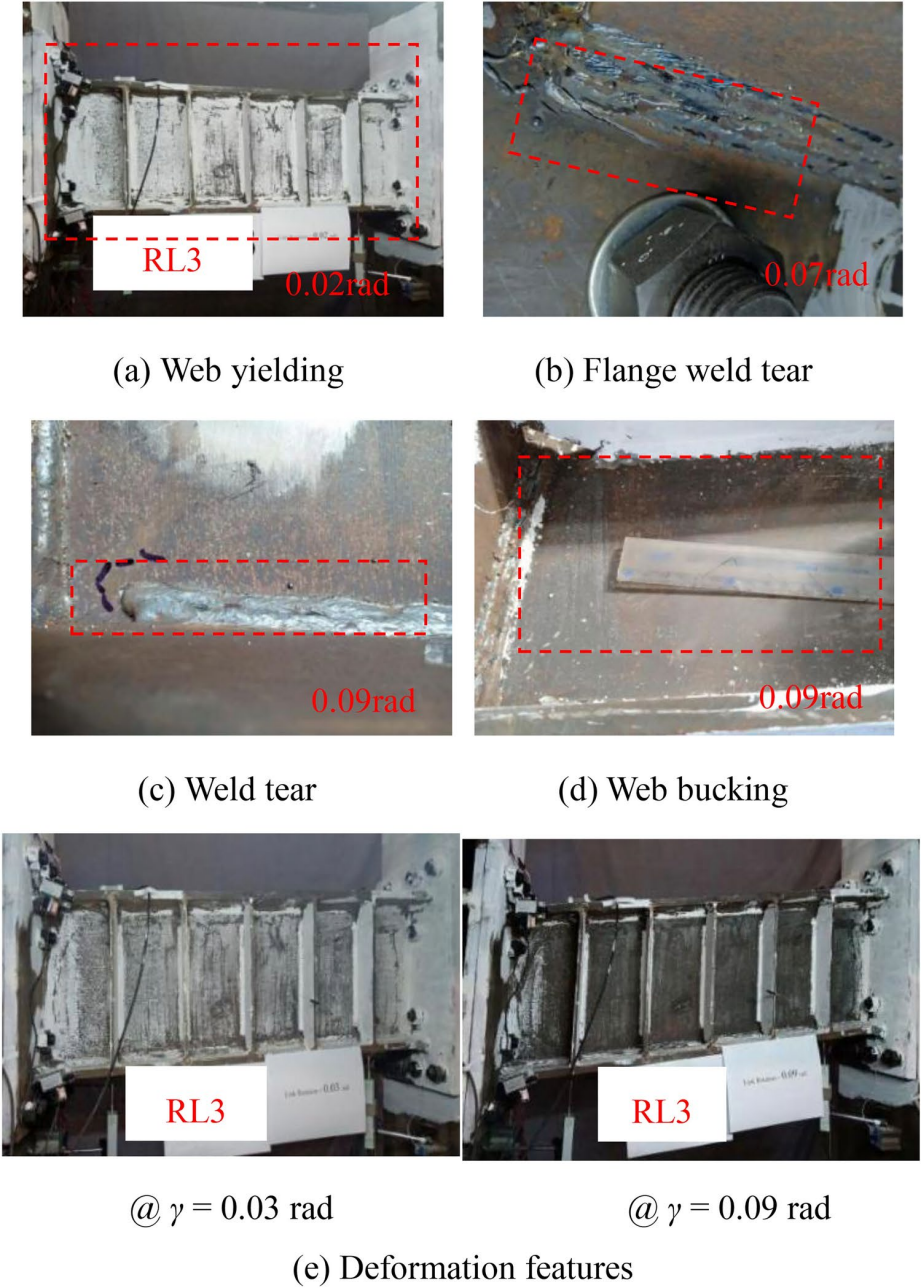
Test phenomena of specimen RL3.

**Fig. 10:**
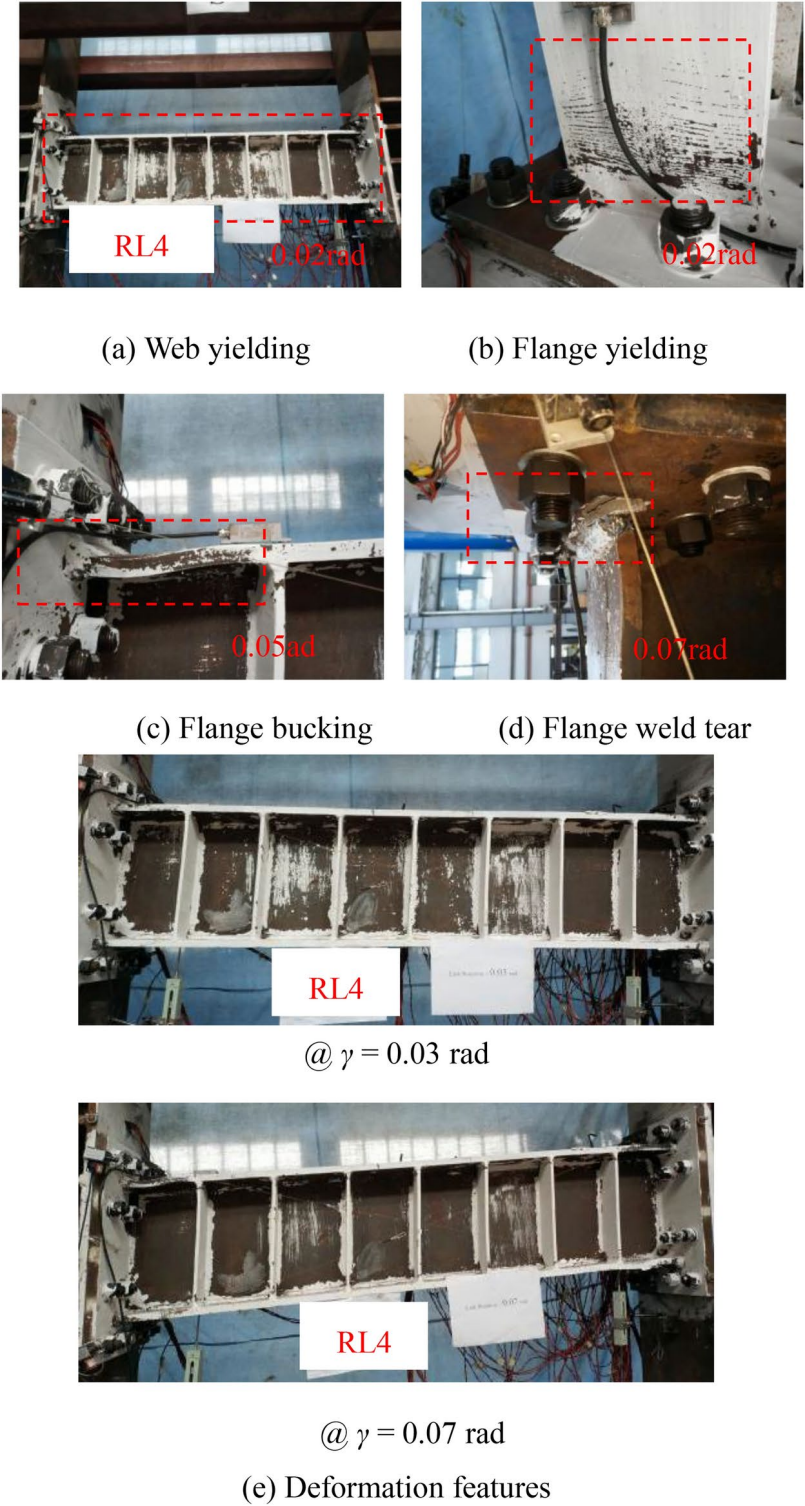
Test phenomena of specimen RL4.

Figures 7 , 8, 9, 10 show the typical failure characteristic and deformation photographs of the specimens. During the cyclic loading test of specimen RL1, web yielding of the link was first observed and other region remained elastic. With the increased displacement, the degree of web yielding significant increased due to the stress hardening effect of web material under cycle loadings; at the same time, the flanges of the steel link began to yield at the completion of cycle 0.02 rad rotation (Figs. 7(a) and 7(b)). When the displacement reached 0.05 rad rotation (Fig. 7(c)), web buckling of the steel link was presented in the center of the web due to the large plastic deformation and high axial constraint by the link end and the intermediate web stiffeners^34^. With the gradual increased loading displacement, degree of the web buckling and web fracture at the corner of the vertical fillet welds in the web-stiffener intersection were further increased. Ultimately, the test was terminated due to the occurrence of obvious web damage (Fig. 7(d)), and the deformation features of damaged specimen also presented in Fig. 7(e).

The deformation and failure characteristics of the specimens RL2 and RL3 were similar to those of the specimens RL1 as shown in Figs. 8 and 9. A slight weld crack in the toe of the flange-to-end plate weld in specimen RL3 was only observed, that of which might affect the replaceable performance of the link but specimens RL1 and specimen RL2 didn’t appear the similar damage. Combined with the deformation and failure characteristics of the links in the test, the specimens RL1 ~ RL3 presented a shear dominated behavior and main damage features included web buckling, web-to-stiffener weld fracture and web tearing. The main reason of weld fracture is that the stress concentrated on the corner of vertical fillet welds was usually presented. In practice, the special details consisting of reduced link sections and end stiffeners might be suggested to alleviate the stress concentration on the fillet weld.

Compared with other three links, specimen RL4 revealed the different failure characteristics as illustrated in Fig. 10. At the beginning of loading stage, web of the specimen RL4 first yielded by the shear behavior. When the displacement reached 0.02 rad rotation, the flange yielding was obvious observed. However, the yield degree and area of the web weakened than those of other specimens with the increased displacement. The damage modes of Specimen RL4 consisted of flange-to-end plate weld crack and flange buckling when the link rotation reached 0.05 rad due to the combined shear-flexural yielding behavior. Finally, the test was terminated because of the occurrence of obvious strength degradation such that the bearing shear strength reduced than 20%. The typical bending-shear failure of specimen RL4 presented the flange-to-end plate weld crack and flange buckling adjusted to the link ends.

Therefore, according to the damage process of these specimens with different short-length ratios, it can be clearly seen that (1) the steel links with short-length ratios resulting in the fully yielded and strain-hardened stress of steel web provided a shear failure mode; (2) there is a general trend that the failure mode of the steel links was gradually changed to that of the specimen with shear-flexure dominated behavior due to the higher value of length ratios; (3) with the increase of link length ratio, the deformation capacity of the steel links might weaken in this test.

### Hysteretic responses

**Fig. 11:**
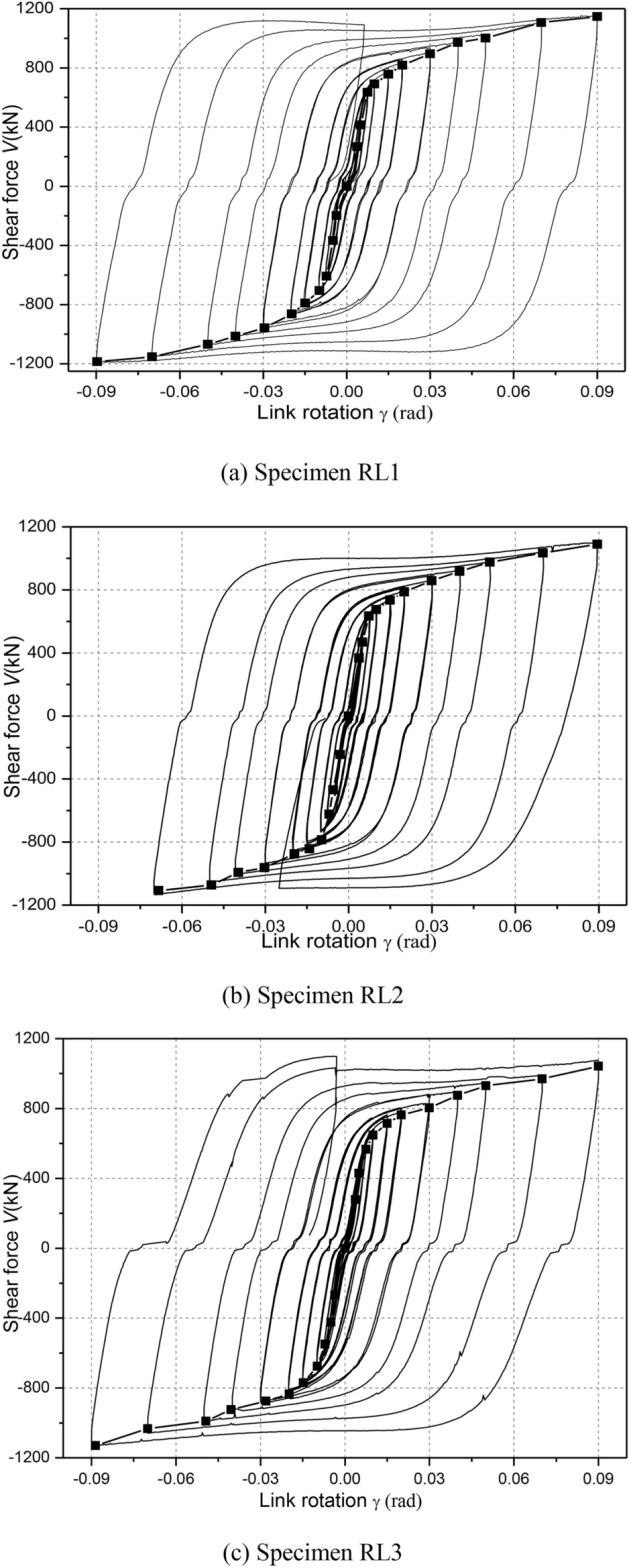

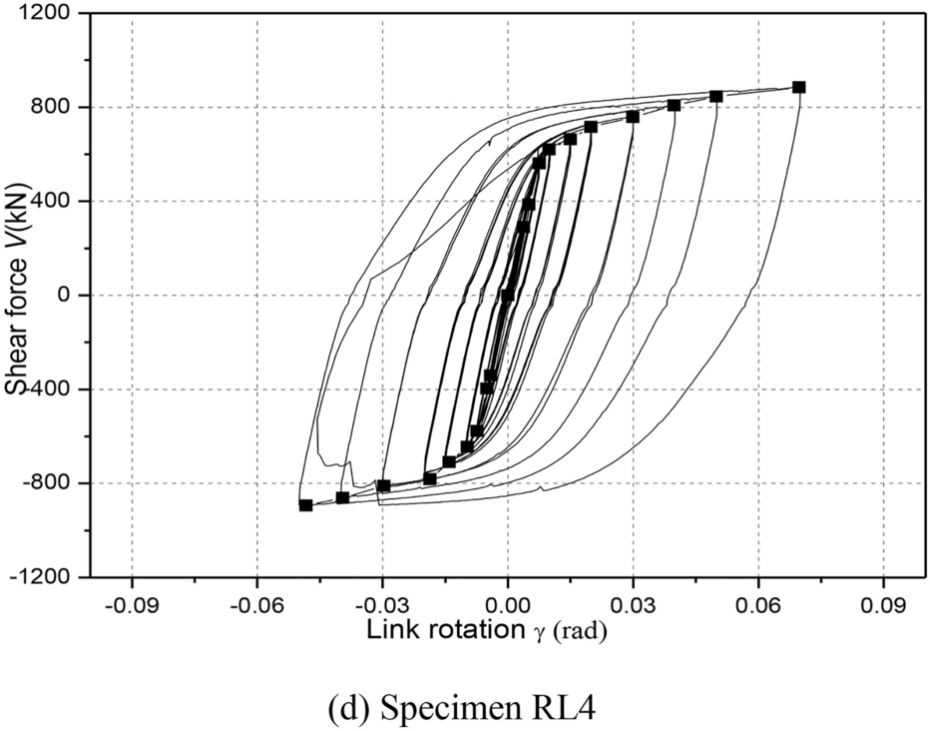
Hysteretic loops.

Figure 11 depicts the hysteretic responses of shear force versus link rotation relationship of each specimen during cyclic loading. Note that link shear force V was calculated as *PH*/*L*, where *P* denotes horizontal load applied by hydraulic actuator (see in Fig. 5), *H* denotes the steel column height and *L* denotes the linked columns spacing, respectively. The link rotation was also calculated as *Lθ*/*e*, where *θ*is the structure inter-story drift angle, that of which referred to some recommendations by Men et al. (2022)^8^.

From these hysteretic curves in Fig. 11, it is clear that all specimens showed very stable hysteretic loops even under link rotation cycles exceeding 0.08 rad, which indicated that the replaceable steel links had good inelastic deformation, excellent energy dissipation and shear bearing capacities. According to the damage process of RL specimens and these hysteretic responses, it can be clearly seen that a slight slippage of the specimens was observed, likely because of a loss of bolt pretension or bolt slipping of specialized link-to-column connections and deconstruction slipping of column-to-floor pins and rigid beam-to-column pins owing to the large deformations.

Furthermore, the shear strengths were obviously strengthened along with an increase in the link rotation before failure. High ductility and deformation capacity of specimens RL1, RL2 and RL3 were fully developed under cyclic loading. Compared with the hysteretic loops of specimens RL1, RL2 and RL3, that of specimen RL4 displayed a slight pinching and a small deformation capacities. The reason was that the nonductile damage at completion of cycle 0.02 rad rotation was appeared and the bending-shear damage feature enlarged continuously until ultimate failure presented mainly due to weaken the cyclic hardening of link web for the specimen with a larger length ratio. These of which resulted in a lower shear strength and smaller deformation capacity.

### Shear strength

**Fig. 12:**
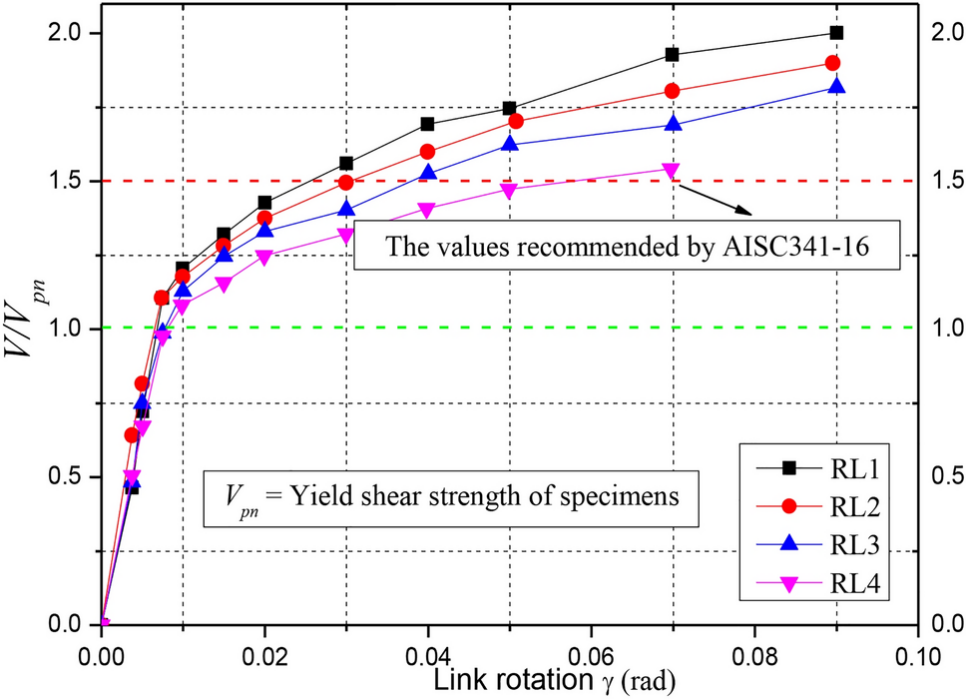
Curves of *V*/*V*_*pn*_ factor and rotation of specimens.

Figure 12 shows the skeleton curves of shear force ratio *V*/*V*_*pn*_ versus link rotation of the specimens under positive loading. The nominal value of shear strength *V*_*pn*_ was calculated as 0.6*f*_*y*_*A*_*w*_from the AISC 341–16 (AISC 2016) provisions^23^, where *f*_*y*_ and *A*_*w*_ denote the measured yield strength of the steel and actual measured section area of link web, respectively. In addition, the value of 1.5 assumed for EBF links in the AISC 341–10 provisions was also provided in Fig. 12 to evaluate the ultimate shear strength.

From the Fig. 12, those curves indicated that the parameter of link length ratio was proved to be vital to the bearing strength of shear links. Before the link rotation reached 0.01 rad, all links had similar initial stiffnesses. With an increasing link rotations, the bearing capacity of each specimen increased until the ultimate loading. The specimens with a length ratio of 0.68 developed a higher overstrength than those with a length ratio of 1.60. The average ratio *V*/*V*_*pn*_of the specimens was 1.9, which is larger than the value of 1.5 specified for EBF links in AISC 341–16 (AISC 2016)^23^. However, shear bearing strength of the specimens with a larger link length ratio was significantly decreased after the web yielded because of the different damage failure counteracted the strain hardening effects during cyclic loading.

## Finite element analysis

In order to develop and validate the observed experimental responses, nonlinear 3D finite element models using ABAQUS 6.14 were also conducted in this paper.

### Finite element modeling

**Fig. 13:**
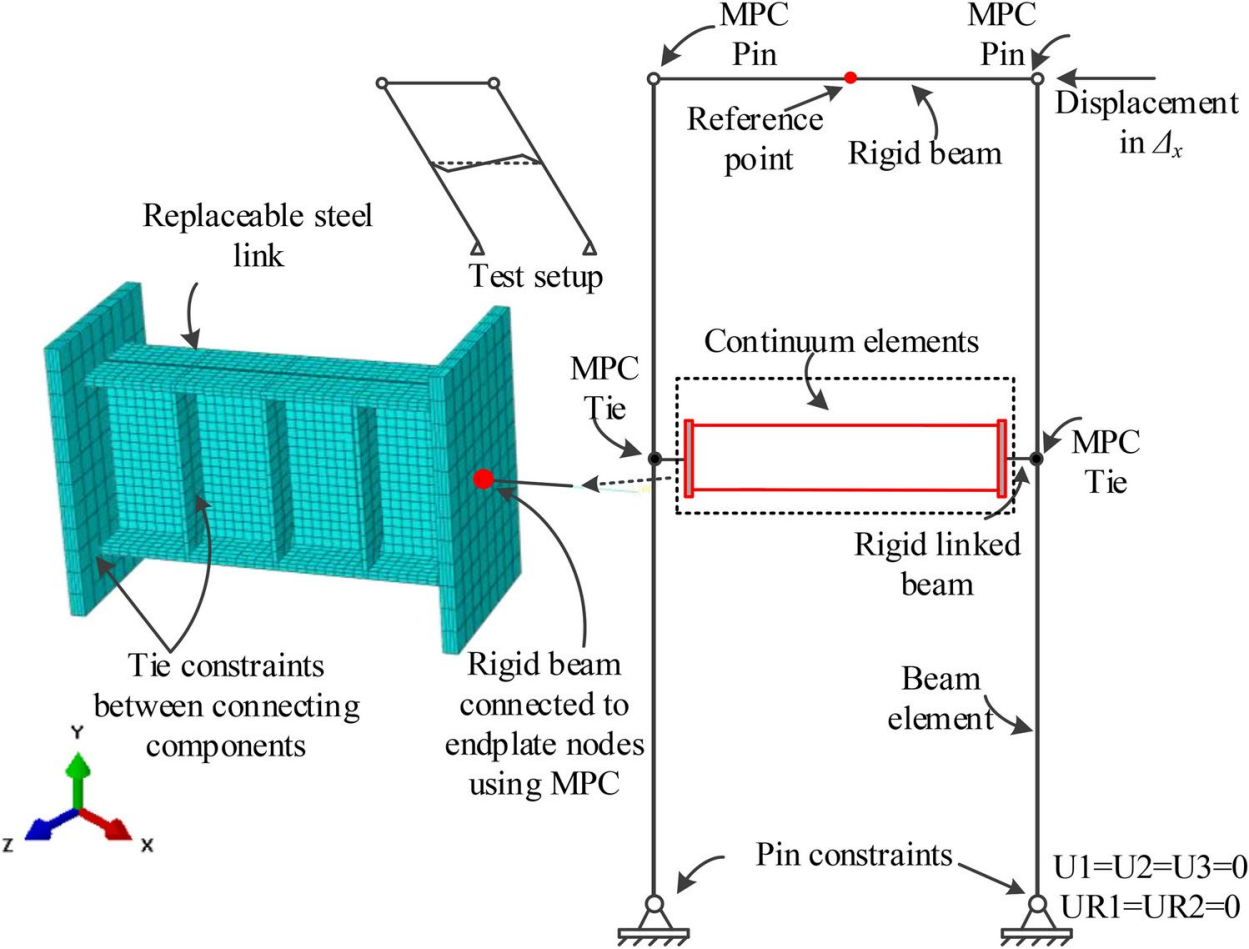
FE model of test specimen.

Figure 13 depicts the nonlinear 3D finite element (FE) model. The FE models consisted of two steel linked columns, replaceable steel links, end plates, rigid beams and stiffeners. To represent the experimental responses and consider computational efficiency, the beam element was used for the steel columns and rigid beam while other structural members adopted to the continuum elements. The geometries of finite element modeling were same as the experimental setup. Additionally, the continuum element were modeled by using the eight-node linear hexahedral solid elements with reduced integration and hourglass control (C3D8R). Multiple point constraints (MPC) were used to connect the steel columns and rigid beam and end plates. Tie constraints were also used between the steel link and end plates and stiffeners. To prevent the out-of-plane deformation of the FE model, reference point was defined in the rigid beam. Besides, two pin constraints were fixed at the bottom of two steel columns.

The Chaboche cyclic constitutive model included a nonlinear isotropic hardening component, and a kinematic hardening component^20,35^was used for the link steel to capture the inelastic cyclic responses of the shear links in the FE models. According to references^35^and^36^, Table 3 shows the calibration parameters of the Q235 and Q345 steel. The elastic modulus *E*_*s*_, yield strength *f*_*y*_ and tensile strength *f*_*u*_ were obtained from the tensile coupon tests. It’s worth noting that both material nonlinearity and geometric nonlinearity were accounted for in this model. Besides, material fracture and tearing were not considered in the FE models.

**Table 3:** Isotropic and kinematic hardening parameters.

Material	Isotropic strengthening parameters			Kinematic hardening parameters					
	σ|_0_ (MPa)	*Q*_*∞*_ (MPa)	*b*	*C*_*1*_ (MPa)	*γ*_*1*_	*C*_*2*_ (MPa)	*γ*_*2*_	*C*_*3*_ (MPa)	*γ*_*3*_
Q235	291	35	2.5	6000	200	5000	130	300	35
Q355	391	21	1.2	7993	175	6773	116	2854	34

### Comparison of hysteretic responses

**Fig. 14:**
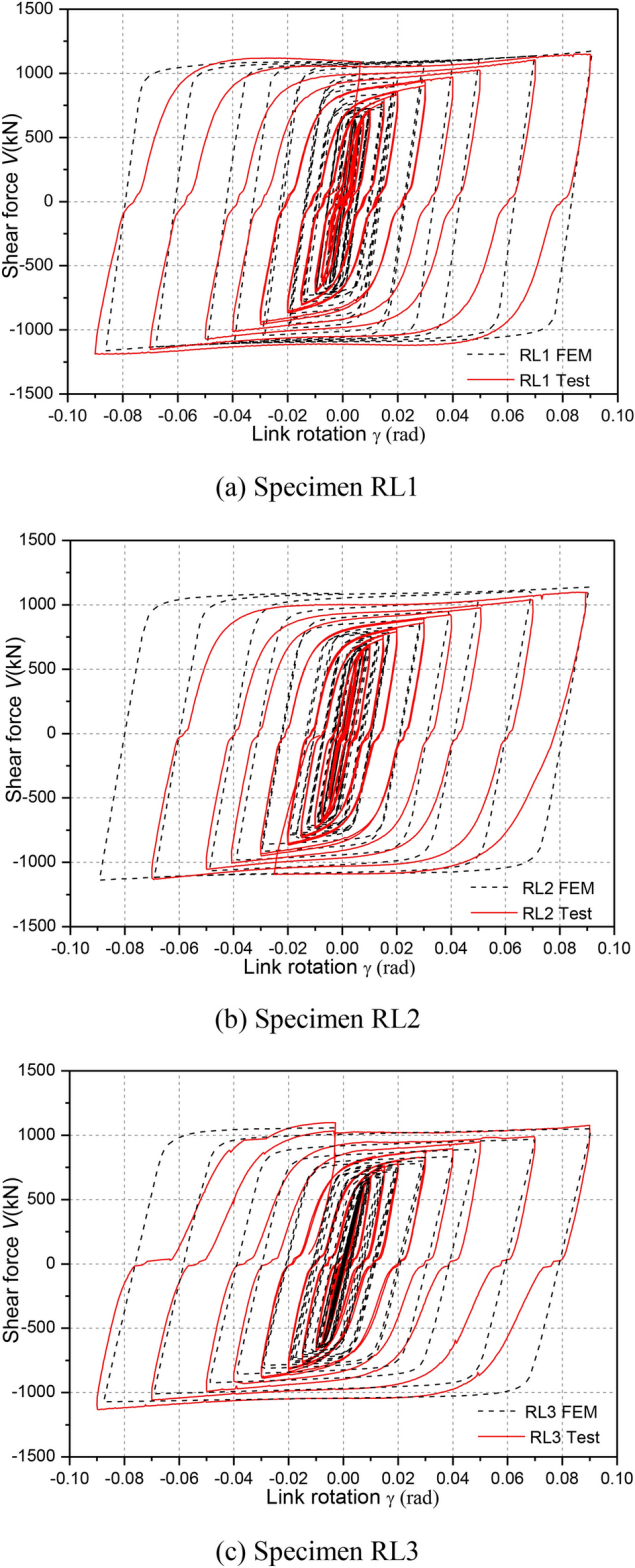

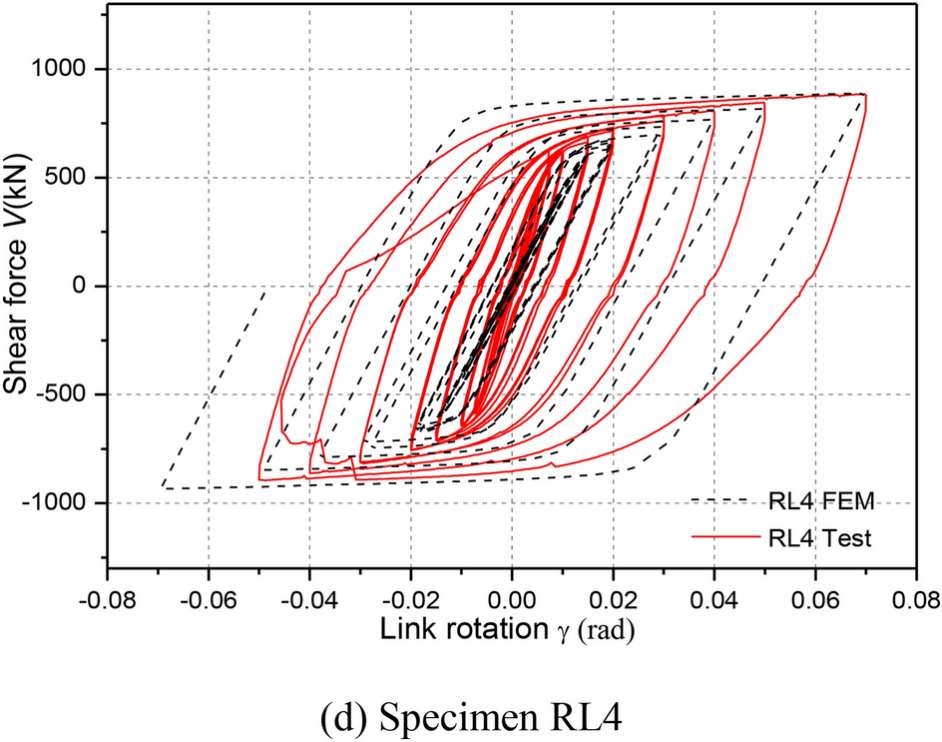
Comparison of hysteretic loops.

Figure 14 shows the comparisons of the FE numerical and experimental hysteretic responses. It can be seen from Fig. 14 that the initial stiffness and shear strength of the FE models at each cycle matched well with the experimental results. The difference of the shear strength values between the FE models and the tests is less than 11%. From Fig. 14, comparisons of the FE numerical and experimental hysteretic responses indicated that the FEMs results do not capture the slip behavior when the stress switches the orientation. The main reason is likely because of the imperfect fabrication or construction tolerance of the bolted connection among these tested structural components owing to the large deformations. Besides, the numerical unloading stiffness for the specimens was slightly lower than the tested values since the insufficient axial constraint effected by the two columns compared to the test setup. Overall, the modeling approach in ABAQUS can capture the cyclic behavior of replaceable steel links accurately.

### Comparison of failure modes

**Fig. 15:**
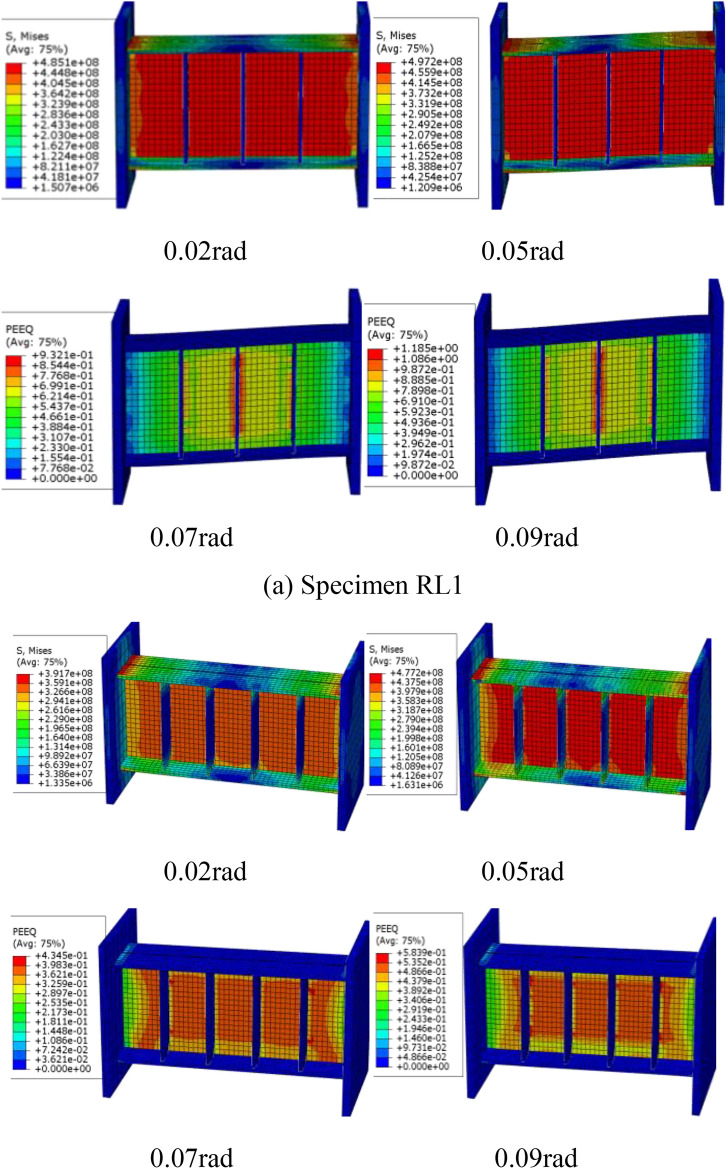

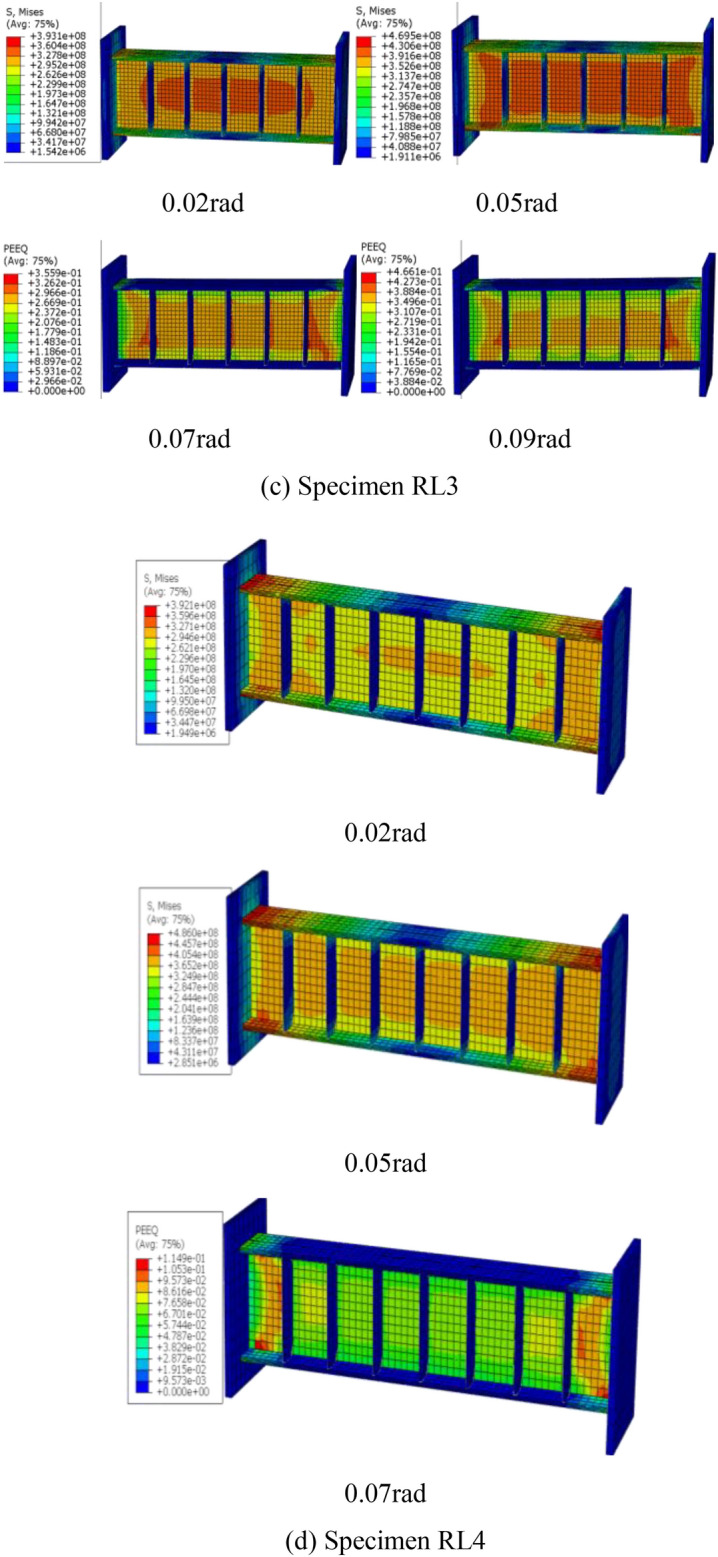
Von Mises stress and PEEQ distributions of the FE models.

According to the typical damage features, Fig. 15exhibits Von Mises stress and PEEQ distributions of the FE models at each cycle of 0.02, 0.05 rad, 0.07 rad and 0.09 rad, respectively. These indexes were generally used to assess the local inelastic strain demand and potential fracture of the steel link^36^. From the Fig. 15, the link web yielding was first observed, which expressed the shear dominated behavior while the difference degree of web yielding presented the effect of short length ratio at 0.02 rad link rotation. When the displacement reached 0.05 rad link rotation, the cyclic hardening effect of web steel was also simulated by the FE modes. In addition, the distribution of the PEEQ value in Fig. 15 mainly concentrated in the web-stiffener intersection and the end plate-flange weld, which indicated that these regions could be damaged under cyclic loading. Note that the FE models can capture the slight web and flange buckling likely due to lack of sufficient axial force by the linked column elements. However, that of which has little effect on the behavior of the local inelastic strain demand and fracture tendency of steel links. Combined with the tested failure modes of these links as previous discussed, the FE models were in good agreement with the experimental results.

Note that, apart from the seismic performance of the replaceable steel links with different short length ratios, limitations and challenges arising from the practical implementation are also described. Firstly, due to the limitation of the experimental and numerical models, the success of the predetermined behavior of the proposed replaceable steel links relies on link behavior, specialized connections, interactive strength between the fuses and other structural members, yield mechanism of each members and so on. Additionally, owners and occupiers, as well as society at large, doubt the architectural function of engineering structures with the replaceable links compared to these traditional systems. Finally, residual deformation for the structure systems with replaceable links might be a critical but often overlooked factor that affects the efficiency of the replacement work in practical engineering. Those parameters of shear steel links in the whole systems on the shear performance and replaceability by the experimental and numerical methods will be acknowledged as an area for future study.

## Conclusions

In this paper, a total of four cyclic loading tests were conducted on the replaceable steel links with different short length ratios used for the earthquake resilient structure. Hybrid sections were used with Q235 steel for the web and Q345 steel for the flanges. The conclusions from this study were drawn as follows:

(1) Compared with the traditional steel or steel–concrete composite frames, this proposed composite frame with replaceable steel links can present a four-level seismic behavior consisting of elastic, rapid return to occupancy, repairable and collapse prevention to achieve recovery of functionality under the action of expected earthquake.

(2) The failure modes of steel links consisted of shear failure and bending-shear failure. The main damage characteristics included web-to-stiffener weld tear, web buckling, and flange-to-end plate weld tear and flange buckling, respectively.

(3) The link specimens presented an excellent bearing capacity, inelastic deformation and energy-dissipating capacity. The specimen RL1 had an inelastic rotation capacity of more than 0.08 rad and an overstrength factor of 2.07, exceeding the values specified for EBF links in AISC 341–10; while the specimen RL4 with a length ratio of 1.60 had an inelastic rotation capacity of 0.5 rad and an overstrength factor of 1.5 due to the bending-shear failure.

(4) Nonlinear FE models of each specimen adopted to different modeling elements were developed using ABAQUS 6.14. Comparison with the hysteresis behavior and failure modes, the FE numerical results were in good agreement with the test results to further evaluate the effects of various parameters on the seismic behavior of this replaceable steel links.

## Data Availability

The datasets used and/or analysed during the current study available from the corresponding author on reasonable request.
